# In-Utero Exposure to Electronic Waste Heavy Metals and Adverse Pregnancy and Neonatal Outcomes: A Systematic Review

**DOI:** 10.3390/ijerph23050665

**Published:** 2026-05-18

**Authors:** Jianna R. D. Sparrow, George Gray, Jordan Fischbach

**Affiliations:** Department of Environmental and Occupational Health, Milken Institute of Public Health, The George Washington University, Washington, DC 20052, USA; gmgray@gwu.edu (G.G.); jordan.fischbach@gwmail.gwu.edu (J.F.)

**Keywords:** electronic waste, heavy metals, prenatal exposure, pregnancy outcomes, neonatal outcomes, fetal growth, informal recycling, biomonitoring

## Abstract

**Highlights:**

**Public health relevance—How does this work relate to a public health issue?**

- Informal electronic waste (e-waste) recycling releases heavy metals that contaminate air, soil, food, and water in nearby communities.
- Pregnant women and developing fetuses are particularly vulnerable to these exposures, raising concerns for adverse pregnancy and neonatal outcomes.

**Public health significance—Why is this work of significance to public health?**

- Electronic waste is the fastest-growing solid waste stream globally and is projected to become one of the most significant environmental and public health challenges of the coming decades.
- This review highlights how toxic metals released from informal e-waste recycling may contribute to adverse pregnancy and neonatal outcomes, underscoring the need to address e-waste as an emerging global health risk.

**Public health implications—What are the key implications or messages for practitioners, policy makers and/or researchers in public health?**

- Strengthening international regulation, formalizing safe e-waste recycling systems, implementing environmental monitoring, and enforcing cradle-to-grave producer responsibility are critical strategies to reduce exposure risks and protect vulnerable populations.
- Expanded epidemiologic research and biomonitoring programs are needed to better understand long-term developmental impacts and guide evidence-based public health interventions.

**Abstract:**

Electronic waste (e-waste) recycling releases heavy metals into surrounding environments, creating potential health risks for nearby populations, particularly pregnant women and developing fetuses. This systematic review evaluated human evidence linking prenatal exposure to heavy metals originating from informal e-waste recycling with adverse pregnancy and neonatal outcomes. Electronic databases, including PubMed and Scopus, were searched through 23 September 2025, for studies measuring heavy metal exposure among pregnant women or neonates living in e-waste–affected communities. Following the Navigation Guide methodology, eight observational studies met the inclusion criteria and were assessed for risk of bias and strength of evidence. Across studies, concentrations of heavy metals were higher in exposed populations and were detected in maternal blood, placenta, cord blood, urine, and meconium samples from exposed populations. Prenatal exposure was consistently associated with adverse outcomes, with many studies reporting statistically significant associations between heavy metal exposure and reduced birth weight, length, head circumference, gestational age, neonatal body mass index, lower Apgar scores, impaired neonatal neurobehavioral development, placental molecular alterations, endocrine disruption, and increased neonatal DNA damage. Overall, the evidence was rated as moderate quality with sufficient evidence linking prenatal heavy-metal exposure from e-waste to impaired fetal growth and neonatal development, and limited evidence for pregnancy complications. These findings highlight the need for improved regulation of e-waste recycling and strengthened public health protections for vulnerable populations.

## 1. Introduction

Electronic waste (e-waste), which includes discarded electronic products such as computers, cell phones, and televisions, is one of the fastest-growing global waste streams. Global e-waste generation reached an estimated 62 million metric tons in 2022, with only 22.3% formally collected and recycled, and is projected to increase to 82 million metric tons by 2030 [1,2]. The remaining e-waste was either informally processed, openly burned, discarded into the environment, or landfilled, creating significant pollution hazards for nearby populations.

Improper disposal of e-waste releases a wide array of toxic substances into surrounding environments, contributing to both acute and chronic health risks. Informal recycling practices—such as open-air burning, acid leaching, manual dismantling, and wire stripping—generate high concentrations of heavy metals and persistent organic pollutants (POPs) that contaminate air, soil, water, and food systems. Heavy metals, including lead (Pb), mercury (Hg), cadmium (Cd), chromium (Cr), arsenic (As), and beryllium (Be), are among the most toxic and persistent contaminants identified at informal e-waste sites [3]. These metals can bioaccumulate through multiple exposure pathways, including inhalation of contaminated air, ingestion of contaminated food or water, and dermal contact, resulting in sustained exposure for both adults and children [4].

Exposure to e-waste-related contaminants disproportionately affects low- and middle-income communities, where informal recycling hubs process a substantial portion of global electronic waste [4]. Environmental sampling studies have reported elevated concentrations of heavy metals in soil, water, and locally produced food near e-waste sites, demonstrating that exposure extends beyond occupational settings to entire communities through environmental and dietary pathways [5,6]. Pregnant women and developing fetuses represent particularly vulnerable populations due to increased physiological susceptibility and critical windows of development. Biomonitoring studies have shown that women residing in e-waste-exposed communities exhibit higher concentrations of heavy metals in maternal blood, urine, and placental tissues compared to reference populations [7,8,9]. These contaminants can cross the placental barrier and have been detected in cord blood and neonatal samples, indicating direct utero exposure [10].

Epidemiological studies have linked prenatal exposure to heavy metals with adverse pregnancy and neonatal outcomes, including reduced birth weight, shortened gestational age, smaller head circumference, and impaired early neurodevelopment [11,12]. In addition to these outcomes, severe complications such as preterm birth, congenital anomalies, and neonatal morbidity have been reported, although findings vary across studies [13,14]. Biologically, heavy metals such as lead, mercury, cadmium, and arsenic act as endocrine disruptors and can introduce oxidative stress, placental dysfunction, and altered nutrient transport, providing mechanistic support for observed epidemiological associations [9,11]. The cumulative effect of such contamination may pose significant risks to human health and raise concerns regarding environmental health protections.

### Rationale for a Systematic Review

Despite a growing body of research evidence, the link between prenatal exposure to e-waste-related heavy metals remains fragmented across study populations, exposure assessments, and outcome measures. Few reviews have specifically focused on pregnant women and neonates, limiting the ability to draw consistent conclusions. This systematic review was conducted to synthesize available human evidence on prenatal exposure to heavy metals from informal e-waste recycling and associated pregnancy and neonatal outcomes. By integrating epidemiological findings and biological mechanisms, this review aims to clarify the consistency and strength of observed associations and identify key gaps for future research and policy development.

## 2. Materials and Methods

### 2.1. Systematic Review Methodology

We conducted the systematic review using the Navigation guide, a systematic review methodology for evaluating environmental evidence based on methods used in the clinical sciences, including the Cochrane Collaboration and Grading Recommendation Assessment, Development and Evaluation (GRADE) framework [15,16].

A preliminary search of PROSPERO was conducted to assess whether similar systematic reviews were underway; no directly comparable registered reviews were identified. This review was not registered in PROSPERO, and a formal protocol was not publicly registered before the study initiation. However, all methodological steps, including inclusion criteria and risk-of-bias domains, were predefined and consistently applied throughout the review process.

### 2.2. Study Question

The objective was to answer the question: “Does prenatal exposure to heavy metals derived from electronic waste increase the risk of adverse pregnancy outcomes or neonatal morbidity and mortality among pregnant women and their offspring?” The “Participants,” “Exposure,” “Comparator,” and “Outcomes” (PECO) statement is briefly outlined below, with additional specifics available in the protocol.

Participants were identified as pregnant cisgender female humans and their neonates (liveborn or stillborn).

Exposure was defined by identifying if the review examined studies of any communities with heavy metal exposures related directly to electronic waste disposal. Exposure was limited to measurements obtained from:

- In utero;
- By placenta;
- Maternal blood;
- Urine during pregnancy/at delivery.

Comparators were identified as pregnant women and neonates not exposed to or with low-risk exposure to heavy metals produced from electronic waste.

Outcomes were defined by any clinical diagnosis of an adverse pregnancy outcome including (a) Preterm birth (<37 weeks’ gestation), (b) Low birth weight (<2500 g), (c) Stillbirth or spontaneous abortion, (d) Intrauterine growth restriction (IUGR), (e) Congenital anomalies as well as any neonatal mortality or morbidity such as (a) Neonatal death (within 28 days of birth), (b) Apgar score reduction (<7 at 5 min), (c) Neonatal jaundice, (d) respiratory distress, (e) distribution of sex hormone levels, and (f) neurodevelopmental delay.

### 2.3. Data Sources

We searched the PubMed and Scopus databases on 23 September 2025, using the search terms listed in Table A1. Following the database search conducted, all retrieved records were downloaded and prepared for abstract and full-text screening. The screening process was conducted through 1 November 2025. We did not limit the search by language or initial publication date. We used the Medical Subject Headings (MeSH) database to compile summaries for adverse pregnancy outcomes and heavy metals produced by electronic waste [16]. We supplemented these results by searching Google Scholar, grey literature databases, and Prospero; consulting with subject matter experts; and hand-searching the references of included studies, review papers on the topic, and references cited by and citing the included studies.

### 2.4. Study Selection

Eligible studies were original human studies that quantified heavy metals from electronic waste in biological samples and assessed associations with adverse pregnancy or neonatal outcomes. Titles and abstracts were screened, and duplicates were removed using RefWorks [17]. Full-text review of potentially eligible studies using predefined inclusion criteria was then carried out. Studies were included if they:

1. Written in English or properly translated;
2. Did mention electronic waste as an exposure or outcome of heavy metal exposure on the study population;
3. It was a direct analysis of heavy metal impact on pregnant or neonatal health outcomes;
4. Written/Published post-1970;
5. The subject was human.

**Figure 1:**
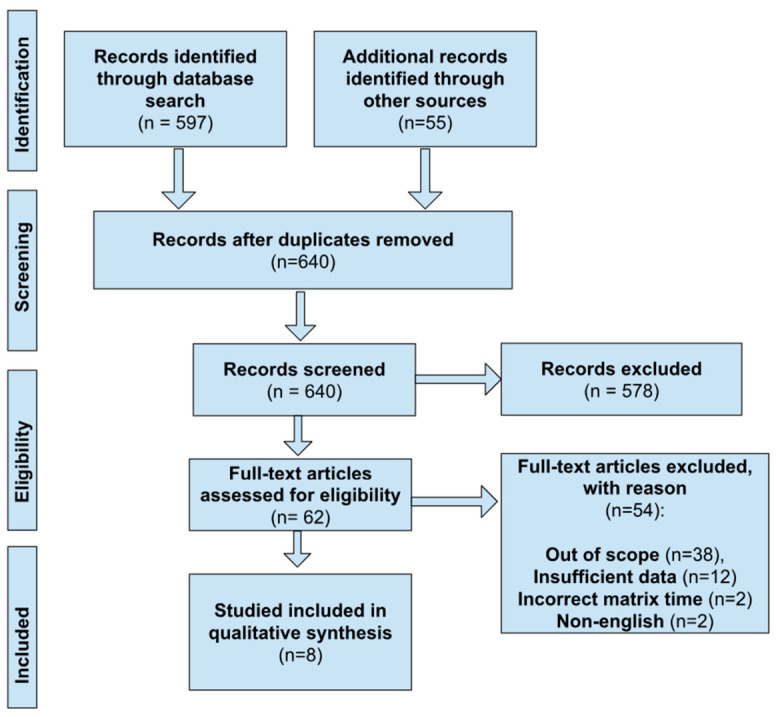
Flowchart—Literature search and screening process.

Studies were excluded if they did not clearly fall into these inclusion guidelines. Study selection was documented using the PRISMA flow diagram (Figure 1). Key study characteristics and effect estimates (e.g., odds ratios, regression coefficients, *p*-values) were extracted using a standardized form.

### 2.5. Rate the Quality and Strength of Evidence

Assessing the risk of bias for each included study. We evaluated the risk of bias for each included study using the Navigation Guide systematic review methodology for rating the quality and strength of the human evidence [16]. Possible ratings for each domain were “low”, “probably low,” “probably high,” or “high” risk of bias, with adapted instructions for each domain based on the type of evidence anticipated beforehand, as shown in Table 1. For example, we determined that for a study to be rated “low” risk of bias for the confounding domain, the analysis must either adjust for all of the following confounders or report that these confounders were evaluated and omitted because inclusions did not substantially affect results: abundance of freshwater fish consumption, maternal age, education, occupation, BMI, gravidity, environmental tobacco smoke exposure, and neonate sex, while several studies also discussed co-pollutant exposures (metals, PAHs, POPs) and socioeconomic or environmental living condition. These confounders were pre-specified in the study protocol due to their well-established potential to influence pregnancy and neonatal outcomes. Examination of outcome reporting across studies did not reveal clear evidence of selective reporting bias. However, variability in exposure biomarkers and outcome definitions limited the ability to assess publication bias formally using statistical methods. Potential reporting bias was evaluated qualitatively by examining whether study outcomes and statistical results appeared selectively reported across studies. Given the limited number of studies and substantial heterogeneity in outcome reporting, formal statistical tests for publication bias (e.g., funnel plot analysis) were not conducted. Instead, patterns of outcome reporting and study findings were examined to identify potential indications of selective outcome reporting.

Assessing the quality of the selected studies. We evaluated the overall quality of the body of evidence across all included studies using the Navigation Guide systematic review methodology [16]. This process involved assessing five primary quality domains: (1) risk of bias across studies, (2) indirectness, (3) inconsistency, (4) imprecision, and (5) publication bias. Each domain was assigned a rating of “no downgrade,” “downgrade,” or “upgrade” based on predefined criteria described in the protocol and adapted from the Navigation Guide and GRADE frameworks, as seen in Table 2.

The initial rating for human observational evidence began as “moderate” quality, consistent with the Navigation Guide approach. We then downgraded or upgraded the body of evidence depending on the presence and magnitude of limitations in each domain. For example, if most studies demonstrated potential residual confounding, exposure misclassification, or outcome measurement error, the quality of the evidence was downgraded one level for “risk of bias.” Conversely, evidence was upgraded if there was a large magnitude of effect, evidence of a dose–response relationship, or if all plausible residual confounding would reduce an observed effect.

Certainty of Evidence. Based on the Navigation Guide framework, the certainty of evidence was evaluated separately for the two primary outcome domains. Evidence linking prenatal exposure to heavy metals from e-waste recycling with adverse pregnancy outcomes was assessed as moderate, reflecting consistent associations across several observational studies despite some limitations in exposure measurement. Evidence linking exposure to neonatal health outcomes was assessed as limited to moderate, due to fewer studies and variability in outcome measures.

Assessing the strength of the body of evidence. Following quality evaluation, we rated the strength of the overall body of evidence according to the Navigation Guide criteria, which includes consideration of the quality rating, direction of the effect estimates, confidence in the effect estimate, and other compelling attributes of the data [16]. Each evidence stream was categorized as “Quality of body of evidence,” “Direction of effect estimate,” “Confidence in effect estimate,” “Other compelling attributes of the data that may influence certainty,” and “Overall strength of evidence,” as seen in Table 3. These ratings reflect the level of certainty that exposure to heavy metals derived from electronic waste contributes to adverse pregnancy or neonatal outcomes. All judgments and rationales were documented in the review’s evidence tables, with supporting notes provided in Table 6. The overall certainty of evidence for each outcome domain was assessed using the Navigation Guide systematic review methodology for environmental health evidence. This framework evaluates the quality, consistency, directness, and precision of available evidence to determine the strength of evidence for each outcome category.

Evidence Synthesis. Due to heterogeneity in exposure assessment methods, biological matrices (e.g., blood, cord blood, placenta, urine), outcome definitions, and statistical reporting across studies, a quantitative meta-analysis was not feasible. When necessary, study results were standardized during data extraction to allow comparison across studies reporting similar outcomes using different statistical metrics. For example, effect estimates reported as regression coefficients, correlations, or odds ratios were interpreted in terms of the direction and magnitude of association rather than being pooled quantitatively. Results from individual studies were summarized using structured evidence tables that describe study design, exposure assessment, outcomes, and reported associations, and risk-of-bias assessments were visualized using summary figures. Instead, results were synthesized using a structured narrative synthesis approach following the Navigation Guide systematic review framework. Studies were grouped according to two primary outcome domains: (1) pregnancy outcomes (e.g., birth weight, gestational age, stillbirth, pregnancy complications) and (2) neonatal health outcomes (e.g., fetal growth indicators and developmental biomarkers). Within each group, study findings were summarized according to exposure type, study design, and direction of reported associations. Sensitivity analyses were not conducted due to the limited number of eligible studies and substantial heterogeneity in exposure measurement and outcome definitions across studies.

## 3. Results

### 3.1. Included Studies

#### 3.1.1. Study Selection

The search retrieved 597 records from PubMed and Scopus, with an additional 55 identified through snowballing and manual reference review. After removing 12 duplicates, 640 unique records were established. Following title and abstract screening, 578 records were excluded for irrelevant exposures not specific to electronic waste and for populations that were not pregnant women or their neonates. A total of 62 full-text articles were assessed for eligibility, and 54 were excluded due to: not meeting PECO criteria (23 had the non-relevant exposure, 11 had the incorrect population, 4 had the incorrect outcome) and 12 had insufficient outcome data, 2 had the wrong sample matrix time, and 2 were non-English articles with no proper translation available. More information on exact reasonings studies that were excluded can be found within the Supplementary Materials. 

Ultimately, 8 studies met the inclusion criteria and were included in the final review (see Figure 1 for PRISMA flow diagram). 

#### 3.1.2. Study Characteristics

The included studies were published between 2007 and 2019 and represented populations located in Guiyu, China, Xiamen, China, Haojiang, China, Chaonan, China, and Shantou, China. Sample sizes ranged from 90 to 24,493 participants, with a median of 220. All included studies were conducted in China, primarily within Guiyu and surrounding regions, indicating a strong geographical concentration of evidence that may limit generalizability to other global e-waste settings.

Study designs included seven cross-sectional comparative studies, a cross-sectional biomarker study, and a retrospective cohort study, and populations focused on pregnant women and their neonates. Exposures primarily involved lead, cadmium, mercury, manganese, PCBs, and metal mixtures assessed through maternal blood, umbilical cord blood, placenta tissue, umbilical cord tissue, umbilical cord venous blood from the placenta, neonatal anthropogenetic measurements, Apgar scores, meconium, neonatal behavioral neurological assessment (NBNA), and maternal urine. Key outcomes measured included birth weight, gestational age, preterm birth, neurodevelopmental indices, hormonal balance, stillbirth, birth length, head circumference, BMI, ponderal index, sex, and lymphocyte DNA damage.

A summary of study characteristics is presented in Table 4 and Table 5.

Across the eight included studies, consistent patterns emerged linking prenatal heavy-metal exposure from e-waste to adverse fetal growth and non-metal outcomes. The most robust and frequently reported findings included reduced birth weight (range: −51 g to −216 g), decreased head circumference (up to −1.96 cm), and impaired neurodevelopmental indicators such as lower Apgar and NBNA scores. Effect estimates were generally directionally consistent across studies, although magnitude varied due to differences in exposure biomarkers, study design, and population characteristics. While odds ratios and regression coefficients were reported using different statistical approaches, the overall direction of effect consistently indicated worse outcomes among exposed populations.

#### 3.1.3. Risk of Bias Assessment for Individual Studies

**Figure 2:**
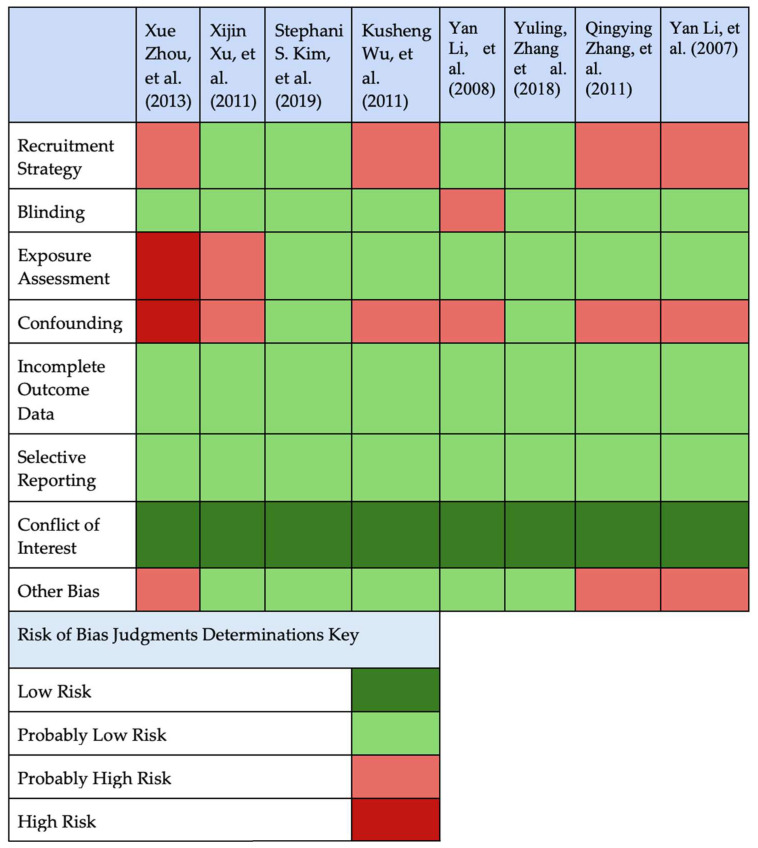
Risk of Bias Judgments [18,19,20,21,22,23,24,25].

Risk of bias was assessed using the adapted checklist shown in Figure 2.

Overall, 0 studies were rated as low risk, 2 as probably low risk, 5 as probably high risk, and 2 as high risk of bias. The most common sources of bias included recruitment strategy (non-random, hospital-based recruitment), limited control for confounding (e.g., socioeconomic status, nutrition, co-exposure), and reliance on area-level exposure data rather than individual-level exposure for some studies.

Studies with a high risk of bias often used small convenience samples, had minimal adjustment for confounders, and defined exposure only by residence in an e-waste area versus a reference town. Whereas studies with lower risk typically used individual biomarker measurements, standardized outcome assessments, clearer eligibility criteria, and multivariable models adjusting for key maternal and neonatal covariates.

A detailed breakdown of bias domains is shown in Table A2.

#### 3.1.4. Quality and Strength of the Body of Evidence

The overall quality and strength of the evidence were evaluated using the U.S. Agency for Healthcare Research and Quality (AHRQ)’s “Strength of Evidence” (SOE) framework. Using the Navigation Guide, we evaluated the overall quality and strength of human evidence examining the relationship between prenatal exposure to heavy metals from electronic waste (e-waste) and adverse pregnancy and neonatal outcomes. In alignment with the Navigation Guide, the human observational evidence begins at a moderate quality rating, and we assessed domains for potential downgrading or upgrading across the eight studies.

Evidence quality for maternal heavy metal e-waste exposure and adverse pregnancy and neonatal outcomes was rated as moderate, supported by the downgraded and upgraded qualities. We downgraded one level for risk of bias, as most of the studies were rated low risk of bias and most were assessed as probably high or probably low risk, primarily due to limitations in recruitment strategy (hospital-based or convenience sampling), incomplete control for socioeconomic and environmental co-exposures, and exposure misclassification resulting from reliance on residence in an e-waste area as a proxy for exposure in some studies. However, nearly all studies directly measured heavy-metal biomarkers in maternal, cord blood, placental, or meconium samples, reducing concerns about exposure misclassification. All other downgrading factors were not considered to be relevant due to all studies addressing the PECO statement, all studies indicating the same direction of effect, and demonstrating statistically significant associations or clear differences between exposed and reference groups. There is a possible inconsistency to note regarding [23]: they found adverse outcomes only in neonatal girls, not in boys. Due to the large effects demonstrated in several studies and two studies reporting dose–response or mixture associations between biomarker concentrations and outcomes, we upgraded the evidence by one level. Therefore, the final quality of evidence rating for human data is moderate.

We rated the overall strength of evidence for the two major outcome groupings: (1) fetal growth & neonatal development, and (2) pregnancy complications.

(1) Fetal Growth & Neonatal Development—Sufficient Evidence of Toxicity

Across the eight studies, there was consistent evidence that prenatal exposure to e-waste–related heavy metals adversely affects fetal growth and neonatal development. All studies measuring anthropometry, gestational duration, Apgar scores, NBNA scores, placental biomarkers, endocrine profiles, or DNA damage found worse outcomes in exposed populations. Associations were coherent, biologically plausible, and supported by multiple biomarkers from different tissue types. Therefore, the strength of evidence supports a causal relationship between prenatal heavy-metal exposure from e-waste and adverse neonatal outcomes.

(2) Pregnancy Complications—Limited Evidence of Toxicity

Only a subset of studies directly evaluated stillbirth, preterm birth, or related pregnancy complications. While the few available studies reported consistently higher rates of stillbirth, low birth weight, and preterm birth in e-waste towns compared with reference communities, the evidence base remains smaller, and residual confounding cannot be excluded. Therefore, the evidence was rated as limited, indicating that the relationship is suggestive but not yet conclusive.

Table 6 summarizes the quality and strength of evidence across domains.

Formal investigations of statistical heterogeneity were not conducted because the included studies varied substantially in exposure biomarkers, study design, and outcome definitions, precluding quantitative comparison. As well, sensitivity analyses were not performed due to the limited number of eligible studies and substantial methodological heterogeneity across exposure measurements and outcome definitions.

Despite overall consistency in the direction of associations, variability in study design, exposure assessment (e.g., biomarker vs. area-level), and outcome definitions limited direct comparability across studies. In addition, residual confounding—particularly from co-exposures (e.g., air pollution, diet, tobacco exposure)—was not consistently controlled for and may influence observed associations.

## 4. Discussion

In this systematic literature review of heavy metal exposure from electronic waste and the effects on pregnant women and their neonates, there were consistent findings across studies conducted primarily in Guiyu, China, and smaller comparable sites in Southeast Asia, demonstrating that prenatal exposure to heavy metals such as lead (Pb), cadmium (Cd), nickel (Ni), and chromium (Cr) from informal e-waste recycling significantly affects fetal and neonatal health outcomes. While consistent associations were observed across studies conducted primarily in Guiyu, China, these findings should be interpreted cautiously given the observational nature of the evidence and potential sources of bias. The studies were consistent with this finding, with statistically significant associations observed between maternal and cord blood metal levels and reductions in neonatal head circumference (β = −0.75 cm, *p* < 0.05), decreased birth weight, disruptions of sex hormones, higher rates of stillbirth, and lower Apgar or neurobehavioral scores. For example, Guiyu neonates exhibited mean cord blood lead concentrations of 113.28 μg/L, compared with 75.45 μg/L in controls, alongside elevated urinary cadmium and hormonal disruption, as reflected by increased estradiol and progesterone levels [21,22,23,24,25,26]. Overall, the findings in the studies examined were consistent that pregnant women and their neonates are impacted by electronic-waste-related heavy-metal exposure.

Across the 8 studies examined in this systematic review, several pregnancy and neonatal outcomes were evaluated repeatedly, enabling comparisons across research groups. Four studies assessed birth weight, all reporting lower mean birth weights among exposed neonates, ranging from −51 g [20] to −216 g [21], with two studies showing statistically significant reductions after adjustment for maternal characteristics and gestational age (e.g., −91 g; 95% CI: −108 to −75). Three studies evaluated head circumference, and all found consistent negative associations, including a −1.96 cm reduction (95% CI: −2.39, −1.52) in exposed infants [20] and significant correlations between maternal urinary cadmium and smaller head circumference among female neonates [24]. Two studies examined the relationship between gestational age and preterm birth, reporting either shorter gestation (e.g., −0.44 weeks [95% CI: −0.66, −0.21]) or increased odds of preterm birth (OR = 1.67; 95% CI: 0.66–4.23), although the effect sizes were imprecise. Neurobehavioral outcomes were assessed in two studies: Li et al. (2008) [22] found significantly lower NBNA behavior subscale scores (10.91 vs. 11.29; *p* = 0.012) and strong inverse correlations between meconium lead and NBNA total score (r = −0.903; *p* < 0.01), while Xu et al. (2011) [19] reported lower Apgar scores among neonates in Guiyu relative to a reference city. One study highlighted the higher rates of stillbirth (OR = 4.20 [3.40–5.18]), underscoring the severity of heavy metal concentrations for neonates [21]. Taken together, these cross-study patterns strengthen the overall evidence by demonstrating that multiple independent research groups consistently identified adverse trends across birth weight, head circumference, gestational age, and early neonatal functioning in populations exposed to e-waste–related metals.

The major drivers for exposure to heavy metals from electronic waste are the open burning of circuit boards, acid leaching for metal recovery, and handling of contaminated dust that releases fine particles containing Pb, Cd, Cr, and other metals [3]. Additional sources include contaminated local food chains, particularly rice and fish cultivated near e-waste sites, and unsafe household storage of dismantled materials. The primary exposure pathways include inhalation of airborne particles, ingestion of contaminated food and water, and dermal absorption during contact with e-waste [3,4,6,8,9,14]. To reduce this exposure in the future, policy and public health measures must focus on formalizing e-waste recycling sectors, implementing occupational-health and safety standards, and strengthening international regulation of electronic waste exports under frameworks like the UN, WHO, and Basel Convention. In addition, health-monitoring programs for pregnant women and children should be established in affected regions, supported by environmental remediation and safer recycling techniques. Short-term actions could include community education campaigns, dust-suppression technology, and air-quality surveillance, while long-term interventions should prioritize circular-economy policies, producer responsibility laws, and investment in certified recycling facilities [3,6,7,9].

The consistency of evidence indicates biologically plausible and statistically supported harm from heavy-metal exposure among pregnant women and their neonates living near informal e-waste recycling zones. More studies should be conducted in other e-waste hotspots worldwide to confirm these associations and explore additional health endpoints such as neurodevelopmental delay, endocrine disruption over the long term, and epigenetic modification [20]. Future work should include multi-site pregnancy cohorts with standardized biomonitoring protocols, mixture-model statistical approaches, and harmonized outcome definitions to strengthen global comparability. Although effect estimates were generally consistent in direction, variation in magnitude and statistical significance across studies suggests heterogeneity in exposure levels, population characteristics, and methodological approaches.

Beyond the studies analyzed, the greater body of literature, including toxicological and mechanistic evidence, supports these associations. Laboratory and epidemiological studies confirm that Pb, Cd, and Cr cross the placental barrier, disrupt endocrine signaling, and impair fetal neurodevelopment, reinforcing the plausibility of observed human outcomes [10].

For our specific review, several strengths were identified, including the careful and deliberate conduct of the review with adequate time allotted for each article. Additionally, this review was based on the Navigation Guide [16], increasing confidence that the systematic review process was accurate and consistent with the recommended process to limit any bias or errors that could occur. Additionally, using both Scopus and PubMed as search tools strengthened the review by opening the search to multiple platforms.

Several limitations occurred, including language barriers, with several studies published only in Chinese. Additionally, all included studies were conducted within a limited geographic region in China, which may reduce the external validity and generalizability of findings to other e-waste-affected regions globally. Another limitation of this review was the lack of additional reviewers, which deviates from the standard practice in scientific literature reviews of having at least two individuals independently screen and assess studies. While only one reviewer conducted the screening and assessment, the findings are considered robust, as the process was closely guided and reviewed throughout, with each stage requiring structured submission and oversight to ensure methodological rigor and consistency. Other constraints included heterogeneous exposure metrics across studies and limited follow-up data to assess long-term child-development outcomes.

An additional limitation of this evidence is the underlying assumption that informal e-waste activity was the predominant or sole source of metal exposure for the mothers and neonates in these studies. However, several concurrent environmental and behavioral exposure pathways during the study periods (2007–2019) could have contributed to elevated heavy-metal levels independent of e-waste recycling. For example, leaded gasoline was still in use—legally in some regions and illegally in others—throughout much of the early 2000s in China, meaning background lead concentrations in air and soil may have been elevated regardless of proximity to e-waste sites [27]. Although several studies attempted to adjust for maternal cigarette smoking, self-report is often unreliable, and secondhand smoke exposure may elevate cadmium and lead levels even when mothers deny active use. Dietary sources, particularly consumption of local fish, shellfish, and rice irrigated with contaminated water, are also established routes of mercury, cadmium, and lead exposure and were not systematically assessed across studies. Consequently, ambient air pollution from traffic, industrial combustion, municipal waste burning, and coal-powered facilities likely contributed additional metal-laden particulates that were not quantified or adjusted for in most analyses. Collectively, these factors underscore that the real-world exposure landscape is multifactorial, and failing to account for these co-exposures may either overestimate or underestimate the specific contribution of informal e-waste recycling. Given that all included studies were observational, causal inferences cannot be definitively established, and the findings should be interpreted as associations rather than confirmed causal relationships. Future studies should therefore integrate environmental monitoring, validated biomarkers, dietary assessments, and spatial exposure modeling to isolate the independent effects of e-waste activity more accurately.

When placed in a broader public health context, these results signal a significant environmental justice crisis: the health costs of global electronic consumption are externalized onto marginalized, low-income recycling communities. Taking what has been learned from this systematic literature review, an assumption could potentially be made for the general health of the public that improper or non-regulated electronic waste recycling is a major environmental hazard that is only continuing to grow [1]. On a population scale, even small downward shifts in average birth weight or head circumference distributions can substantially increase the proportion of infants falling below clinical thresholds, compounding morbidity and economic burden for already resource-limited regions. These findings highlight that unregulated e-waste dismantling exposes entire communities, including women of reproductive age and those currently pregnant, to neurotoxic and endocrine-disrupting metals through contaminated soil, air, and water [6]. These results show measurable changes in birth outcomes, and several studies highlighted the need for deeper investigation into long-term developmental and potential epigenetic risks among exposed populations, given the observed adverse infant associations and their potential to create lasting intergenerational impacts.

Although causality cannot be fully established, the evidence supports immediate precautionary action. Policy recommendations include banning transboundary dumping of e-waste, investing in circular-economy infrastructure, promoting manufacturer take-back programs, encouraging the use of technological devices until the end of their life, and providing healthcare screening and intervention programs for vulnerable populations [27]. While the findings highlight important public health concerns, policy implications should be interpreted cautiously and in the context of the available evidence base. While the current evidence suggests a consistent pattern of adverse associations, additional research across diverse populations and settings is needed to confirm these findings and strengthen their generalizability. If these findings continue to be supported, countries continuing to contribute to e-waste exploration should bear financial responsibility for remediation and health protection in affected areas. Other recommendations to be considered based on the findings could be establishing a centralized formal recycling facility equipped with air-filtration and acid-recovery systems and worker PPE, combined with a maternal-and-neonatal biomonitoring initiative and public education campaign to reduce exposure and promote sustainable livelihoods.

Every pregnant woman should have the right to eat and drink safely within her own community, without fear that global consumption patterns and waste mismanagement are endangering her child. Yet in many e-waste-affected regions, women are involuntarily exposed to hazardous substances through contaminated environments, occupational activities, and a lack of regulatory protection. The issue represents not only a public health crisis but also a profound ethical and social justice concern.

These findings are consistent with broader environmental health literature demonstrating that prenatal exposure to heavy metals such as lead and cadmium is associated with impaired fetal growth, neurodevelopmental deficits, and endocrine disruption. However, given the current state of epidemiological evidence and the limited data available from communities outside of Guiyu, China, additional studies are needed to further assess the impacts of e-waste exposure on pregnant women and neonates, and to evaluate the consistency, external validity, and generalizability of these findings across diverse populations. Future research should prioritize identifying trimester-specific susceptibility, evaluating multi-metal interactions, and incorporating intervention effectiveness studies to close remaining knowledge gaps.

## 5. Conclusions

In this systematic review using the Navigation Guide, we concluded that there was consistent evidence that heavy metals from e-waste sites have negative effects on pregnant women and their neonates. The findings suggest that heavy metals impact the birth weight, gestational age, neurobehavioral outcomes, and stillbirth rates for affected populations compared to non-exposed control groups. Intervention approaches would benefit from updated methods and strategies to promote a healthier, more sustainable way of recycling electronic waste. Future research should be done to continue to emphasize the impact on pregnant women and neonates in these communities while also studying other locations of e-waste recycling to be able to generalize these findings outside of Southeast China. However, changes in the practice of e-waste disposal could impact this research, but all impacts associated with less adverse outcomes would be considered a positive change in recycling techniques. The consistency of evidence across multiple biological matrices and outcomes underscores the urgency of regulating global e-waste flows and investing in safe recycling systems. Protecting pregnant women and their infants from this exposure is both a scientific and moral imperative that requires coordinated international action and further research. These findings emphasize that improving global e-waste governance is essential to protect maternal and neonatal health.

## Figures and Tables

**Table 1:** Instructions for Making Risk-of-Bias Determinations.

Risk of Bias Domain	Low Risk of Bias Designation
Recruitment strategy	Participant recruitment protected against selection bias
Blinding	Knowledge of exposure is prevented when assessing outcome
Exposure Assessment	Risk of exposure misclassification is minimized through validated methods
Confounding	Analysis adjusted for fresh water fish consumption, maternal age, education, occupation, BMI, gravidity, environmental tobacco smoke exposure, neonate sex, co-pollutant exposures (metals, PAHs, POPs) and socioeconomic or environmental living condition
Incomplete outcome data	Any missing outcome data is not likely to introduce bias
Selective outcome data	All outcomes specific in method have been reported
Conflict of interest	Study free of support from individual or entity having financial interest in outcome of study
Other bias	Study appears to be free of other sources of bias

Assumed if the review does not meet “low risk” then it would be a degree of “probably low”, “probably high” or “high”.

**Table 2:** Factors for evaluating the quality of the body of human evidence.

Evaluation Factors	Summary of Criteria
Downgrading factors	
Risk of bias	Study limitations include a substantial risk of bias across the body of evidence
Indirectness	Evidence was not directly comparable to the question of interest [i.e., PECO]
Inconsistency	Estimates of effect in similar populations were widely different (heterogeneity or variability in results)
Imprecision	Studies included few participants and few events (wide Cls)
Publication Bias	Studies were missing from body of evidence, resulting in an over-or underestimate of true effects from exposure
Upgrading factors	
Large magnitude of effect	The rating was upgraded if modeling suggested that confounding alone was unlikely to explain associations that were judged to be of large magnitude
Dose response	The rating was upgraded if the relationship between dose and response in one or multiple studies and/or the dose response across studies were consistent
Confounding minimizes effect	The rating was upgraded if the consideration of all plausible residual confounders or biases would underestimate the effect or suggest a spurious effect when results show no effect

**Table 3:** Factors for evaluating strength of evidence.

Evaluation Factors	Summary of Criteria
Quality of body of evidence	Overall confidence in the collective studies’ design, conduct, and limitations
Direction of effect estimate	Consistency and coherence in whether the effect estimates across studies suggest increased, decreased, or no association between exposure and outcome. Consistent findings across multiple independent studies strengthen the evidence
Confidence in effect estimate	Degree of certainty that the observed association reflects a true causal relationship rather than chance, bias, or confounding. Determined by study quality, precision, and reproducibility of results
Other compelling attributes of the data that may influence certainty	Additional features that increase certainty in the evidence, such as biological plausibility, coherence with mechanistic or animal evidence, or consistency across populations and study designs
Overall strength of evidence	Judgment considering all factors above classified as one of the following categories: “Sufficient evidence,” “Limited evidence,” “Inadequate evidence,” “Evidence of lack of toxicity”

**Table 4:** Summary of Study Characteristics (pt.1).

Author, Date	Source/Study	Study Population	Location	Sample Size	Sample Period	Sample Matrix
Xue Zhou, et al. (2013) [18]	Disruption of sex hormones and oxidative homeostasis in parturient women and their matching fetuses at an e-waste recycling site in China	46 pregnant women and their neonates residing in e-waste site and 44 pregnant women and their neonates in reference town; full-term pregnancies, no hormone therapy	Guiyu, China for exposed population, city 50 km away for referents	Total: 90, Exposed Population: 46, Referent Population: 44	December 2005–July 2006	Maternal blood, umbilical cord blood, placenta, and umbilical cord tissue
Xijin Xu, et al. (2011) [19]	Birth outcomes related to informal e-waste recycling in Guiyu, China	All birth records obtained from the local hospital archives for women living in Guiyu and all birth records from the Xiamen Maternal and Child Health Hospital for women living in suburban and rural areas of Xiamen	Guiyu, China for exposed population, Suburban and rural area of Xiamen for un-exposed population	Retrospective: Total: 24,493 registered births, Exposed: 4094, Un-exposed: 20,399, Cord Samples: Total: 531, Guiyu: 432, Xiamen: 99	Retrospective: 2001–2008 Cord Samples: 2004–2009	Retrospective cohort study with birth records & umbilical cord venous blood from the placenta
Stephani S. Kim, et al. (2019) [20]	Birth outcomes associated with maternal exposure to metals from informal electronic waste recycling in Guiyu, China	Pregnant women 18 years and older with a singleton pregnancy, lived in their town for the duration of their pregnancy and consented to participate in the study, women were excluded if they had history of psychiatric or thyroid disorders, women were studied from Guiyu (exposed population) and Haojiang (un-exposed population)	Guiyu, China (e-waste site) and Haojiang, China (unexposed to e-waste)	Exposed: *n* = 314 Unexposed: *n* = 320	2013?	Electronic neonate-weighing scale to measure birth weight, a calibrate length board for birth length, and a measuring tape for head circumference as well as maternal blood samples
Kusheng Wu, et al. (2011) [21]	In utero exposure to polychlorinated biphenyls and reduced neonatal physiological development from Guiyu, China	Healthy pregnant women were recruited from hospitals in Guiyu (exposed) and Chaonan (unexposed)	Guiyu, China (exposed to e-waste) and Chaonan district of Shantou city, China (un-exposed)	167 participants (108 from Guiyu and 59 from Chaonan)	May and July of 2007	UCB samples and neonatal physiological index (height, weight, body mass index, gestational age, and Apgar score)
Yan Li, et al. (2008) [22]	Monitoring of lead load and its effect on neonatal behavioral neurological assessment scores in Guiyu, an electronic waste recycling town in China	Full-term neonates from the Department of Gynecology and Obstetrics of the local hospital in Guiyu, China and full-term neonates from the Department of Gynecology and Obstetrics of Shantou Chaonan Minsheng Hospital	Guiyu, China (exposed to e-waste) and Chaonan district of Shantou city, China (un-exposed)	152 total participants (100 from Guiyu and 52 from Chaonan)	July–October 2006	Cord blood samples, meconium (first stool of the newborn) and NBNA (Neonatal Behavioral Neurological Assessment) collected by trained nurses
Yuling Zhang, et al. (2018) [23]	Maternal urinary cadmium levels during pregnancy associated with risk of sex-dependent birth outcomes from an e-waste pollution site in China	Mother-neonate pairs were recruited before birth from Guiyu township and Haojiang District	Guiyu, China (exposed to e-waste) and Haojiang District (un-exposed)	449 total subjects, 237 from Guiyu and 212 from Haojiang	September 2011 to June 2012	Maternal urine samples, Apgar scores, and gestational age, birth weight, birth length, birth BMI, head circumference
Qingying Zhang, et al. (2011) [24]	Downregulation of placental S100P is associated with cadmium exposure in Guiyu, an e-waste recycling town in China	Pregnant women registered to give birth at hospitals in Guiyu and Shatou	Guiyu, China (exposed to e-waste) and Shantou, China (un-exposed)	105 pregnant women, 55 from Guiyu and 50 from Shantou	October 2008–June 2009	4 full-depth samples of placental tissue from the central region of each placenta and maternal age, gestational age, infant length, infant weight, Apgar score, sex
Yan Li, et al. (2007) [25]	The hazard of chromium exposure to neonates in Guiyu, China	Full-term neonates from the Department of Gynecology and Obstetrics of local hospital of Guiyu and full-term neonates from the Department of Gynecology and Obstetrics of Shantou Chaonan Minsheng Hospital	Guiyu, China (exposed to e-waste) and Shantou Chaonan, China (un-exposed)	100 neonates from Guiyu and 52 neonates from Shantou Chaonan with an additional 150 umbilical cord blood samples from neonates (100 from Guiyu and 50 from Shantou) → total at end of study: 302 cases of neonates (200 from Guiyu and 102 from Shantou)	July to October 2006 for neonates and 2007 for umbilical cord samples	Cord blood of neonates and 5 mL of umbilical cord blood collected from the placenta. Physical characteristics: neonates body length, body mass, Apgar scores, gender, gestation age, and delivery type (normal labor/cesarean delivery)

**Table 5:** Summary of Study Characteristics (pt.2).

Author, Date	Measurement Timing	Quantitative Relationship	Qualitative Associations	Outcome
Xue Zhou, et al. (2013) [18]	Blood samples collected upon admission for delivery; cord and placental samples collected immediately post-delivery	Compared e-waste-exposed vs. reference groups for sex hormones (E2, PROG, TESTO), mRNA expression (ERα, ERβ, PR), and oxidative stress biomarkers (MDA, SOD, GPx)	Positive correlations: Maternal and cord E2 (r = 0.271, *p* < 0.05); MDA, SOD, GPx between maternal and cord sera (r = 0.902, 0.867, 0.850, *p* < 0.01). Negative relationships: SOD/GPx activities decreased while MDA increased in exposed subjects.	Elevated estradiol and progesterone levels; up-regulation of ERα and ERβ mRNA and down-regulation of PR mRNA; increased MDA and reduced SOD and GPx activities → disruption of sex hormone balance and oxidative homeostasis in pregnant women and fetuses exposed to e-waste.
Xijin Xu, et al. (2011) [19]	Cord samples: Shortly after delivery	Compared e-waste-exposed vs. reference group for adverse birth outcomes (stillbirths, low birth weight, Term LBW, twin birth rate, and CBPb levels	Regression findings: Guiyu had ≈4× higher risk of stillbirth (OR = 4.20 [3.40–5.18]); lower mean birth weight (–91 g [–108 to –75]); higher LBW (OR = 1.61 [1.37–1.90]) and term-LBW (OR = 2.12 [1.68–2.69]). Median CBPb: 10.78 µg/dL in Guiyu vs. 2.25 µg/dL in Xiamen (*p* < 0.01). Correlations: CBPb positively associated with maternal/paternal e-waste work and years residing in Guiyu (r = 0.25–0.41, *p* < 0.001).	Prenatal exposure to informal e-waste recycling is associated with higher rates of stillbirth, LBW, term-LBW, lower Apgar scores, and unsafe cord-blood lead levels in newborns → indicating adverse fetal growth and neonatal outcomes linked to maternal e-waste exposure.
Stephani S. Kim, et al. (2019) [20]	Immediately after delivery	Modeling approach: Multiple linear regression of birth outcomes on ln-transformed maternal whole-blood metals (Pb, Cd, Cr, Mn), mutually adjusted and controlling for maternal age, education, occupation, pre-pregnancy BMI, gravidity, environmental tobacco smoke, and infant sex; logistic regression for SGA and preterm; BKMR for mixture effects. Site comparison (exposed Guiyu vs. control Haojiang): adjusted mean differences estimated for GA, BW, length, HC, BMI, PI.	Lead (Pb): lower Head circumference β = −0.75 cm (−1.17, −0.32); lower Ponderal Index β = −0.62 kg/m^3^ (−1.13, −0.11). Cadmium (Cd): lower BMI β = −0.21 kg/m^2^ (−0.41, −0.01); trend lower Birth weight β = −56 g (−116, 5). Higher SGA risk: OR = 2.10 (0.997–4.43). Chromium (Cr): Trend ↓ Head circumference β = −0.28 cm (−0.60, 0.05) (NS). Manganese (Mn): Non-significant ↑ BMI/HC; lower SGA risk: OR = 0.34 (0.16–0.72). Mixture (BKMR): Monotonic decrease in PI as Pb + Cd + Cr + Mn co-increase from 25th to the 75th pct; cumulative negative effects also seen for HC and BMI; no cumulative effect on BW.	Gestational age: +0.44 weeks (0.21, 0.66) in Guiyu. Birth weight: −51 g (−132, 29) (not significant). Birth length: +1.17 cm (0.83, 1.52). Head circumference: −1.96 cm (−2.39, −1.52). BMI: −0.77 kg/m^2^ (−1.03, −0.51). Ponderal Index: −2.01 kg/m^3^ (−2.54, −1.47). Preterm birth: OR = 1.67 (0.66, 4.23) (not significant). Small for gestational age: OR = 1.17 (0.57, 2.40) (not significant)
Kusheng Wu, et al. (2011) [21]	UCB samples from placenta after delivery, neonatal physiological index after delivery and Apgar score (1) 1 min after birth, (2) 5 min after birth and potentially (3) 10 min after birth	Exposure measurement: 28 PCB congeners in umbilical cord blood (UCB) by GC–MS; concentrations lipid-adjusted (ng/g lipid). Design/Comparisons: Guiyu (e-waste) vs. Chaonan (control); statistics included t/χ^2^ tests, Pearson correlations of congeners with neonatal indices, and multiple linear regression to identify predictors of ΣPCBs (mother’s e-waste work, home used as workshop, age). Exposure contrast (ΣPCBs): Median 338.56 ng/g lipid (Guiyu) vs. 140.16 ng/g lipid (Chaonan)	Site-level neonatal score: Lower Apgar in Guiyu (*p* ≈ 0.001). Congener–outcome correlations (Pearson r, *p*): CB-105: Apgar r = −0.174 (*p* < 0.05); BMI r = −0.186 (*p* < 0.05) CB-114: Height r = −0.190 (*p* < 0.05); Apgar r = −0.199 (*p* < 0.05); BMI r = −0.196 (*p* < 0.05) CB-138: Weight r = −0.294 (*p* < 0.05); BMI r = −0.325 (*p* < 0.05) CB-153: Weight r = −0.297 (*p* < 0.05); Apgar r = −0.203 (*p* < 0.05); Gestational age r = −0.322 (*p* < 0.05) CB-180: Height r = −0.278 (*p* < 0.05); Weight r = −0.299 (*p* < 0.05); Gestational age r = −0.497 (*p* < 0.01) Adverse outcomes & exposure: Higher ΣPCBs in adverse birth outcomes (preterm, LBW, stillbirth) vs. normal births (*p* ≈ 0.03). Determinants of ΣPCBs (regression): Higher levels with mother’s e-waste work and house used as workshop; age also retained (model adj. R^2^ ≈ 0.32).	Evidence of reduced neonatal physiological development with higher prenatal PCB exposure: lower Apgar, and inverse associations with height, weight, BMI, and gestational age for several congeners; elevated ΣPCBs among adverse birth outcomes.
Yan Li, et al. (2008) [22]	Post delivery for cord blood samples and meconium with 24 h. of birth, NBNA third day after delivery (48 to 72 h after birth)	Design & sample: Cross-sectional comparison of Guiyu (exposed) neonates (*n* = 100) vs. neighboring control town (*n* = 52). Exposure metrics: Umbilical cord blood lead (CBPb) and meconium lead (MPb) by GFAAS. Neurodevelopment metric: Neonatal Behavioral Neurological Assessment (NBNA) (total score + behavior and active-tone subscales). Statistics: Group comparisons (t/Z tests); Spearman correlations between CBPb/MPb and NBNA; correlations of maternal e-waste–related factors with CBPb/MPb	Lead levels (means): CBPb: Guiyu 113.28 vs. control 60.43 (units as reported; *p* < 0.05 after log-transform). MPb: Guiyu 2.50 vs. control 1.20 (units as reported; *p* < 0.001 after log-transform). NBNA group differences: Total NBNA: Guiyu 38.46 vs. control 38.92 (Z = −2.023, *p* = 0.043). Behavior subscale: Guiyu 10.91 vs. control 11.29 (Z = −2.511, *p* = 0.012). Active tone: NS (Z = −0.870, *p* = 0.385). Exposure–effect correlations: MPb vs. NBNA total: r = −0.903, *p* < 0.01; behavior: r = −0.826, *p* < 0.01; active tone: r = −0.637, *p* < 0.01. CBPb vs. NBNA: no significant correlation (*p* > 0.05). Determinants of exposure: Maternal e-waste work, residence in Guiyu, time living/visiting e-waste sites during pregnancy, and paternal e-waste work were positively correlated with CBPb/MPb (rs ≈ 0.17–0.38, *p* ≤ 0.05)	Neonates from the e-waste town had significantly higher lead loads and lower NBNA performance, with strong inverse correlations between meconium lead and neurobehavioral scores, indicating impaired neonatal neurobehavior associated with in-utero lead exposure from informal e-waste recycling.
Yuling Zhang, et al. (2018) [23]	Maternal urine samples were collected at 7 a.m.–8 a.m. on the day of delivery. APGAR scores were taken after delivery at 1 min and 5 min, all other measurements right after delivery	Design & sample: Cross-sectional comparison of mother–neonate pairs in Guiyu (*n* = 237) vs. Haojiang (*n* = 212). Exposure: Maternal urinary cadmium (U-Cd, µg/g creatinine) measured at delivery by GFAAS; creatinine-adjusted. Modeling: Sex-stratified linear regression of birth outcomes on U-Cd, adjusted for maternal age, weight, height, pre-pregnancy BMI, and education; Mann–Whitney U for U-Cd group differences; Spearman correlations for determinants of U-Cd.	U-Cd levels: Significantly higher in Guiyu than Haojiang; e.g., medians (µg/g Cr) approximately 0.92–1.59 in Guiyu vs. 0.59–0.67 in Haojiang (sex-specific strata), *p* < 0.001. Determinants of exposure: Higher U-Cd with residence in Guiyu (rs ≈ 0.28, *p* < 0.001), closer to the e-waste site/highway, and lower income (positive correlation). Sex-specific regression results (adjusted): Females: Higher U-Cd associated with lower birth weight (*p* = 0.036), shorter birth length (*p* = 0.036), smaller head circumference (*p* < 0.001), and lower Apgar at 1 min (*p* = 0.002) and 5 min (*p* < 0.001). Males: No significant associations for anthropometry after adjustment; Apgar 1-min was lower with higher U-Cd (*p* = 0.004)	Maternal Cd exposure (U-Cd) in the e-waste area is linked to adverse neonatal outcomes predominantly among girls (smaller weight, length, head circumference, and reduced Apgar scores) with limited effects in boys (Apgar 1-min only). Findings support sex-dependent vulnerability to prenatal Cd in e-waste-impacted settings.
Qingying Zhang, et al. (2011) [24]	Placenta tissue samples were collected immediately after delivery; characteristics were collected after birth and Apgar scores were done once at 1 min and once at 5 min after birth	Design & sample: Cross-sectional comparison of placentas from Guiyu (*n* = 55) vs. Shantou (*n* = 50). Exposures (placenta): Cadmium (PCCd) and lead (PCPb) via GFAAS. Biomarkers: S100P (mRNA by RT-qPCR; protein by Western/IHC) and metallothionein (MT) (protein by IHC). Stats: Group comparisons (t/Mann–Whitney U), Spearman correlations of metals with S100P/MT and with maternal/infant characteristics	Exposure contrast (median, ng/g placenta): PCCd 83.99 (IQR 54.10–149.84) vs. 51.59 (30.10–81.90), *p* < 0.001; PCPb 521.01 vs. 273.24, *p* = 0.299 (NS). S100P downregulation (Guiyu vs. Shantou): mRNA (relative units): 0.175 vs. 1.462, *p* < 0.001. Protein (IOD): 0.026 vs. 0.033, *p* = 0.045. MT upregulation (IOD): 0.058 vs. 0.038, *p* = 0.003. Metal–biomarker correlations: PCCd lower S100P protein: r = −0.262, *p* = 0.008. PCCd higher MT protein: r = 0.241, *p* = 0.016. PCPb with S100P/MT: NS (r = −0.079, *p* = 0.432; r = 0.137, *p* = 0.179). Other correlations: PCCd higher with longer maternal/paternal residence in Guiyu (r ≈ 0.21, *p* ≈ 0.04–0.05) and paternal smoking (r = 0.212, *p* = 0.040); Apgar slightly lower with higher PCCd (r = −0.195, *p* = 0.050)	Guiyu placentas show higher cadmium and a marked decrease in S100P (with compensatory higher MT), indicating a cadmium-associated placental stress response. Lead in the placenta was not linked to S100P/MT changes. Infant anthropometrics were similar between towns; the borderline inverse link between PCCd and Apgar suggests potential functional impact but requires confirmation.
Yan Li, et al. (2007) [25]	Directly after delivery for cord blood samples, body length, body mass, APGAR scores (once at 1 min and once at 5 min), gender, gestation age, and delivery type (vaginal labor/cesarean delivery)	Design & sample: Cross-sectional comparison of neonates from Guiyu (e-waste town; 2006 *n* = 100, 2007 *n* = 100) vs. Chaonan controls (2006 *n* = 52, 2007 *n* = 50). Exposure: Umbilical cord blood chromium levels (UCBCLs, μg/L) by GFAAS. Biological effect endpoint: DNA damage in cord-blood lymphocytes via alkaline comet assay (injury rate % cells with tails; tail length μm). Statistics: Group contrasts (M–U tests, *t*-tests/χ^2^), Spearman correlations of UCBCLs with exposure determinants and comet metrics.	Exposure contrast (UCBCLs): 2006: Guiyu mean 306.2, median 93.89 μg/L vs. control mean 19.95, median 18.10; Z ≈ −8.44, *p* < 0.01. 2007: Guiyu mean 99.90, median 70.60 μg/L vs. control mean 32.48, median 24.00; Z ≈ −8.08, *p* < 0.01. No significant change in Guiyu 2006→2007 (Z ≈ −0.999, *p* > 0.05). Determinants of higher UCBCLs (Spearman rs): residence in Guiyu (rs 0.68–0.51, *p* < 0.01), longer residence/time in Guiyu during gestation (0.65–0.44, *p* < 0.01), maternal e-waste work (0.20–0.35, *p* ≤ 0.01), paternal e-waste work (0.27–0.42, *p* < 0.01), workshop residence (0.19, *p* = 0.02), maternal smoking (0.22, *p* = 0.01), time roaming exposed sites (0.23, *p* < 0.01). Anthropometry vs. UCBCLs: No significant correlations with neonatal length or weight in either year (*p* > 0.25–0.94). DNA damage (comet assay): Injury rate (% cells with tail): Guiyu 33.2% vs. control 10.7% (χ^2^ = 343.47, *p* < 0.01). Tail length (μm): Guiyu 4.49 ± 1.92 vs. control 2.09 ± 0.65 (τ = 6.78, *p* < 0.01). Dose–effect: UCBCLs correlated strongly with injury rate (rs = 0.95, *p* < 0.01) and tail length (rs = 0.89, *p* < 0.01).	Neonates from the e-waste town had significantly elevated cord-blood chromium and significantly greater lymphocyte DNA damage; DNA damage increased monotonically with UCB chromium. No measurable association with birth size was detected. Findings indicate substantial prenatal chromium exposure from informal e-waste recycling with genotoxic effects at birth.

**Table 6:** Summary of findings, quality of evidence, strength of evidence.

Quality Factor	Rating	Basis
Downgrade		
Risk of bias across studies	−1	All eight studies were observational, and none were rated low risk of bias. Most had probably high or high risk in recruitment, confounding, and exposure misclassification. However, many used biomonitoring of metals, which partially mitigated concerns.
Indirectness	0	All studies directly evaluated pregnant women or neonates in e-waste communities, using heavy metals known to originate from e-waste as exposures, and reported clinically meaningful pregnancy or neonatal outcomes. There were no PECO mismatches.
Inconsistency	0	The direction of effect was consistent across all eight studies. All studies found worse outcomes in e-waste-exposed groups.
Imprecision	0	Statistically significant differences or clear effect directions were reported in most studies.
Publication bias	0	No evidence suggesting selective reporting. Studies came from multiple research groups across different years with varying outcomes and biomarkers.
Upgrade		
Large magnitude of effect	+1	Reports of large, meaningful differences in most studies. These magnitudes exceed what could be explained by bias alone.
Dose response	+1	Two studies showed mixture effects or dose–response associations between biomarker levels and decreasing neonatal anthropometry or NBNA scores.
Confounding minimizes effect	0	Although unmeasured or residual confounding was present across studies, the direction of confounding would likely bias findings toward the null rather than exaggerate harm. Many exposed and reference communities were socioeconomically similar, and several confounders were partially controlled
Overall quality of evidence	Moderate	Human observational evidence begins at moderate quality per Navigation Guide criteria. One downgrade was applied for risk of bias, but two factors justified upgrading: (1) large magnitude of effect across multiple studies, and (2) dose–response relationships. After accounting for all domains, the final determination remained moderate quality.
Summary of qualitative findings	Across included studies, heavy-metal exposures originating from informal e-waste recycling (lead, cadmium, chromium, nickel, manganese, PCBs) were consistently higher among pregnant women and neonates living in e-waste–exposed communities. All studies reported adverse pregnancy or neonatal outcomes associated with these exposures. Findings included reduced birth weight, length, gestational age, and head circumference; lower Apgar scores and neonatal behavioral neurological assessment scores; elevated oxidative stress and sex-hormone disruptions; placental toxicity and S100P downregulation; and increased neonatal DNA damage. No study showed evidence of protective effects or null associations in the opposite direction. The qualitative review supports a coherent and biologically plausible pattern indicating that prenatal exposure to e-waste-related heavy metals harms fetal growth and early neonatal development, with suggestive evidence of increased risk of pregnancy complications.	
Strength Considerations		
Quality of body of evidence	Moderate	Human observational studies started at moderate quality. One downgrade was applied for risk of bias, and one upgrade was applied for large effect sizes and dose–response relationships, maintaining a final moderate quality rating.
Direction of effect estimate	Consistent	Across all eight studies, the effect estimates consistently indicated adverse effects of heavy-metal exposure from e-waste on fetal growth, neurodevelopment, placental function, oxidative stress, and pregnancy outcomes. No study showed protective or null effects in the opposite direction.
Confidence in effect estimate	Moderate-High	Despite some confounding and exposure-misclassification concerns, confidence is strengthened by repeated findings across independent studies; use of objective biomarkers (placenta, cord blood, maternal blood, urine, meconium); mixture analyses; and statistically significant or large effect estimates in several studies.
Other compelling attributes of the data that may influence certainty	Large effects, dose–response	Several studies reported large magnitudes of effect (e.g., ~4× increased stillbirth risk, reduced head circumference, high cord-blood lead). Two studies demonstrated dose–response relationships, and mechanistic support was provided through endocrine disruption, placental S100P reduction, oxidative stress, and DNA damage. These factors increase certainty that the association is real and causal.
Overall strength of evidence	Sufficient (adverse neonatal outcomes) & Limited evidence (adverse pregnancy outcomes)	The totality of evidence supports a causal relationship between prenatal heavy-metal exposure from e-waste and adverse neonatal outcomes. For pregnancy complications such as preterm birth and stillbirth, the evidence is limited due to fewer studies, but still suggestive of harm.

## Data Availability

No new datasets were generated during this study. All data analyzed in this review were extracted from previously published studies cited within the manuscript. Evidence tables and data extraction materials used in this review are available from the corresponding author upon reasonable request.

## Supplementary Materials

The following supporting information can be downloaded at: https://www.mdpi.com/article/10.3390/ijerph23050665/s1, Table S1: Describing Databases; Table S2: Excluded Studies and Reasonings. File S1: PRISMA_2020_abstract_checklist [28]; File S2: PRISMA_2020_checklist [28].

## Abbreviations

The following abbreviations are used in this manuscript:

AHRQ	Agency for Healthcare Research and Quality
BMI	Body Mass Index
BKMR	Bayesian Kernel Machine Regression
CBPb	Cord Blood Lead
Cd	Cadmium
CI	Confidence Interval
Cr	Chromium
DNA	Deoxyribonucleic Acid
E2	Estradiol
E-waste	Electronic Waste
GFAAS	Graphite Furnace Atomic Absorption Spectrometry
GPx	Glutathione Peroxidase
IUGR	Intrauterine Growth Restriction
LBW	Low Birth Weight
MDA	Malondialdehyde
Mn	Manganese
NBNA	Neonatal Behavioral Neurological Assessment
Ni	Nickel
OR	Odds Ratio
PAHs	Polycyclic Aromatic Hydrocarbons
Pb	Lead
PCBs	Polychlorinated Biphenyls
PECO	Participants, Exposure, Comparator, Outcome
POPs	Persistent Organic Pollutants
PRISMA	Preferred Reporting Items for Systematic Reviews and Meta-Analyses
RT-qPCR	Reverse Transcription Quantitative Polymerase Chain Reaction
SOE	Strength of Evidence
UCB	Umbilical Cord Blood
UCBCLs	Umbilical Cord Blood Chromium Levels
U-Cd	Urinary Cadmium

## Appendix A

**Table A1:** Search Terms.

Search	PubMed
Population Terms—Pregnant Women & Neonates	(women [mesh] OR “reproductive age” [mesh] OR “Adolescent” [mesh] OR “Young Adult” [mesh] OR Adult [mesh] OR “pregnant women” [tiab] OR “childbearing age women” [tiab] OR “women of reproductive age” [tiab] OR “reproductive-aged women” [tiab] OR “women 15–49 years” [tiab] OR “fertile women” [tiab] OR “maternal age” [tiab] OR “periconceptional women” [tiab] OR “birthing person” [tiab] OR gravid [tiab] OR gestating [tiab] OR “expecting parent” [tiab])
Exposure Terms—E-Waste Heavy Metals	(“heavy metal” [tiab] OR “heavy metals” [tiab] OR “e-waste” [tiab] OR “e-wastes” [tiab] OR “electronic waste” [tiab] OR “electronic wastes” [tiab] OR “developmental neurotoxicants” [tiab] OR scavenging [tiab] OR “dumping on land” [tiab] OR “dumping in water bodies” [tiab] OR “landfilling along with regular waste” [tiab] OR “open burning” [tiab] OR “open heating” [tiab] OR “acid baths” [tiab] OR “acid leaching” [tiab] OR “manual disassembly” [tiab])
Outcome Terms—Adverse Pregnancy Outcomes & Neonate Morbidity/Mortality	(“pregnancy complications” [mesh] OR “Obstetric Labor Complications” [mesh] OR “Pregnancy Complications, Cardiovascular” [mesh] OR “Pregnancy Complications, Infectious” [mesh] OR “Pregnancy Complications, Hematologic” [mesh] OR “Pregnancy Complications, Neoplastic” [mesh] OR “pregnancy complication*” [tiab] OR “obstetric complication*” [tiab] OR “maternal complication*” [tiab] OR “antenatal complication*” [tiab] OR “gestational complication*” [tiab] OR “preeclampsia” [tiab] OR “gestational diabetes” [tiab] OR “preterm labor” [tiab] OR “preterm birth” [tiab] OR “placenta previa” [tiab] OR “placental abruption” [tiab] OR “intrauterine growth restriction” [tiab] OR “maternal morbidity” [tiab] OR “maternal mortality” [tiab] OR miscarriages [tiab] OR “low birth weight” [tiab] OR premie [tiab])
Search	Scopus
Population Terms—Pregnant Women & Neonates	(women [mesh] OR “reproductive age” [mesh] OR “Adolescent” [mesh] OR “Young Adult” [mesh] OR Adult [mesh] OR “pregnant women” [tiab] OR “childbearing age women” [tiab] OR “women of reproductive age” [tiab] OR “reproductive-aged women” [tiab] OR “women 15–49 years” [tiab] OR “fertile women” [tiab] OR “maternal age” [tiab] OR “periconceptional women” [tiab] OR “birthing person” [tiab] OR gravid [tiab] OR gestating [tiab] OR “expecting parent” [tiab])
Exposure Terms—E-Waste Heavy Metals	(“heavy metal” [tiab] OR “heavy metals” [tiab] OR “e-waste” [tiab] OR “e-wastes” [tiab] OR “electronic waste” [tiab] OR “electronic wastes” [tiab] OR “developmental neurotoxicants” [tiab] OR scavenging [tiab] OR “dumping on land” [tiab] OR “dumping in water bodies” [tiab] OR “landfilling along with regular waste” [tiab] OR “open burning” [tiab] OR “open heating” [tiab] OR “acid baths” [tiab] OR “acid leaching” [tiab] OR “manual disassembly” [tiab])
Outcome Terms—Adverse Pregnancy Outcomes & Neonate Morbidity/Mortality	(“pregnancy complications” [mesh] OR “Obstetric Labor Complications” [mesh] OR “Pregnancy Complications, Cardiovascular” [mesh] OR “Pregnancy Complications, Infectious” [mesh] OR “Pregnancy Complications, Hematologic” [mesh] OR “Pregnancy Complications, Neoplastic” [mesh] OR “pregnancy complication*” [tiab] OR “obstetric complication*” [tiab] OR “maternal complication*” [tiab] OR “antenatal complication*” [tiab] OR “gestational complication*” [tiab] OR “preeclampsia” [tiab] OR “gestational diabetes” [tiab] OR “preterm labor” [tiab] OR “preterm birth” [tiab] OR “placenta previa” [tiab] OR “placental abruption” [tiab] OR “intrauterine growth restriction” [tiab] OR “maternal morbidity” [tiab] OR “maternal mortality” [tiab] OR miscarriages [tiab] OR “low birth weight” [tiab] OR premie [tiab]

## Appendix B

**Table A2:** Risk of Bias Rubric with Reasoning.

Bias Category	Rating	Reasoning
Xue Zhou, et al. (2013) [18]		
Recruitment Strategy	Probably high	Small hospital-based convenience samples in two towns
Blinding	Probably low	Laboratory assays of hormones/oxidative markers. No knowledge of exposure while analyzing.
Exposure Assessment	High	Exposure defined only by residence in e-waste vs. residence in non-e-waste; no actual pollutant measurements
Confounding	High	No adjustment for SES, smoking, nutrition, etc.
Incomplete Outcome Data	Probably low	No loss of follow up and delivery-based study
Selective Reporting	Probably low	No evidence of selective non-reporting but no pre-registered protocol
Conflict of Interest	Low	Explict “no conflicts of interest”
Other Bias	Probably high	Small sample, many biomarkers, no direct exposure data, no direct date of experiment
Xijin Xu, et al. (2011) [19]		
Recruitment Strategy	Probably low	Census of 24,493 births from official records in two regions
Blinding	Probably low	Birth outcomes from routine records and outcome assessors not aware of the hypothesis
Exposure Assessment	Probably high	Main exposure is living in Guiyu vs. Xiamen; cord-blood Pb only on a subset
Confounding	Probably high	Limited individual-level covariates reported
Incomplete Outcome Data	Probably low	No loss of follow up and record-based study
Selective Reporting	Probably low	No evidence of selective non-reporting but no pre-registered protocol
Conflict of Interest	Low	Explict “no conflicts of interest”
Other Bias	Probably low	Potential differences for care and record quality between cities
Stephani S. Kim, et al. (2019) [20]		
Recruitment Strategy	Probably low	Clearly defined inclusion/exclusion; consecutive enrollment at delivery and IRB approval
Blinding	Probably low	Objectives measures and standardized anthropometry and lab assays
Exposure Assessment	Probably low	Multi-metal maternal blood biomarkers with validated methods
Confounding	Probably low	Adjusts for age, education, occupation, pre-pregnancy BMI, gravidity, and sex.
Incomplete Outcome Data	Probably low	No loss of follow up and delivery-based study
Selective Reporting	Probably low	No evidence of selective non-reporting but no pre-registered protocol
Conflict of Interest	Low	Explict “no conflicts of interest”
Other Bias	Probably low	Measurement error acknowledged
Kusheng Wu, et al. (2011) [21]		
Recruitment Strategy	Probably high	Hospital-based sample with limited detail on participation rates
Blinding	Probably low	Standard neonatal measures
Exposure Assessment	Probably low	Validated PCB in umbilical cord blood methods
Confounding	Probably high	Correlation/regression with minimal covariate adjustment
Incomplete Outcome Data	Probably low	No loss of follow up and delivery-based study
Selective Reporting	Probably low	No evidence of selective non-reporting but no pre-registered protocol
Conflict of Interest	Low	Explict “no conflicts of interest”
Other Bias	Probably low	Limited reporting on potential co-exposures
Yan Li, et al. (2008) [22]		
Recruitment Strategy	Probably low	Limited recruitment detail
Blinding	Probably high	NBNA scoring partly subjective; assessors likely aware of town status
Exposure Assessment	Probably low	Cord blood and meconium Pb measured in lab
Confounding	Probably high	Non multivariable adjustment despite potential SES and environmental confounders
Incomplete Outcome Data	Probably low	No loss of follow up and delivery-based study
Selective Reporting	Probably low	No evidence of selective non-reporting but no pre-registered protocol
Conflict of Interest	Low	Explict “no conflicts of interest”
Other Bias	Probably low	Small sample, single town vs. control
Yuling Zhang, et al. (2016) [23]		
Recruitment Strategy	Probably low	Flowchart, clear numbers and two-town recruitment
Blinding	Probably low	Lab measurements of U-Cd; anthropometry and Apgar by trained nurses unaware of hypothesis
Exposure Assessment	Probably low	Urinary at delivery and creatine-corrected
Confounding	Probably low	Adjusts for maternal age, weight, height, BMI, education, smoking, alcohol, diet, e-waste variables.
Incomplete Outcome Data	Probably low	No loss of follow up and delivery-based study
Selective Reporting	Probably low	No evidence of selective non-reporting but no pre-registered protocol
Conflict of Interest	Low	Explict “no conflicts of interest”
Other Bias	Probably low	Reasonable sample size, clear protocol, good lab methods.
Qingying Zhang, et al. (2011) [24]		
Recruitment Strategy	Probably high	105 women recruited at delivery from two towns: convenience-like
Blinding	Probably low	Blinding not explicit but unlikely differential
Exposure Assessment	Probably low	Cd and PB measured in placenta
Confounding	Probably high	Placental Cd/S100P relationships evaluated largely without confounder control
Incomplete Outcome Data	Probably low	No loss of follow up and delivery-based study
Selective Reporting	Probably low	No evidence of selective non-reporting but no pre-registered protocol
Conflict of Interest	Low	Explict “no conflicts of interest”
Other Bias	Probably high	Limited generalizability and no direct health outcomes listed
Yan Li, et al. (2007) [25]		
Recruitment Strategy	Probably high	Two town hospital samples across two years, limited info on non-participants
Blinding	Probably low	Lab Cr assays
Exposure Assessment	Probably low	UCB chromium measured objectively
Confounding	Probably high	Group comparisons with limited covariate control
Incomplete Outcome Data	Probably low	No loss of follow up and delivery-based study
Selective Reporting	Probably low	No evidence of selective non-reporting but no pre-registered protocol
Conflict of Interest	Low	Explict “no conflicts of interest”
Other Bias	Probably high	Potential temporal changes in exposure not fully handled
