# Supplementary material for: In-Utero Exposure to Electronic Waste Heavy Metals and Adverse Pregnancy and Neonatal Outcomes: A Systematic Review
Source: Int J Environ Res Public Health. 2026 May 18;23(5):665. doi: 10.3390/ijerph23050665 (PMC13206999; doi:10.3390/ijerph23050665)
Supplement: Supplementary file 1 [file ijerph-23-00665-s001.zip › ijerph-4220689-supplementary tables.pdf]

Supplemental Materials for *In-Utero Exposure to Electronic Waste Heavy Metals and Adverse Pregnancy and Neonatal Outcomes: A Systematic Review*

**Table S1.** Describing Databases			
**Database Name**	**Description**	**Search Dates**	**Hit Rates**
PubMed	A free database of biomedical and life sciences literature launched in 1996 by NCBI and NLM and run by the National Institutes of Health.	September 23, 2025,	243
Scopus	Large database that includes comprehensive global content for relevant research by top experts and independent reviews launched in 2004 by Elsevier.	September 23, 2025,	354
Snowballing	*Taken from document found in PubMed named "Health consequences of exposure to e-waste: a systematic review" written by Kristen Grant, et al.*	*September 23, 2025,*	*55*

**Table S2.** Excluded Studies and Reasonings			
**Citation**	**Reason(s) excluded**	**Flowchart Category**	**PECO Specific**
Wu, K., Xu, X., Peng, L., Liu, J., Guo, Y., & Huo, X. (2012). Association between maternal exposure to perfluorooctanoic acid (PFOA) from electronic waste recycling and neonatal health outcomes. Environment International, 48, 1–8. 10.1016/j.envint.2012.06.018	PFOAs not heavy metals	PECO	exposure: PFOAs
Bowring, J. (2005). Heavy metal toxicity and the unborn child. Midwifery Today with International Midwife, (76)(76), 48, 67.	Heavy metal exposure on unborn child; can be used as evidence of effect but not an exposure outcome analysis	PECO	outcome
Wang, Z., Hang, J. G., Feng, H., Shi, L. L., Dong, J. J., Shen, B., Luo, T., Cai, R. M., Shen, L. J., Kido, T., & Sun, X. L. (2019). Effects of perinatal dioxin	6 months and 3 years post-delivery, unfamiliar sample matrix	Sample matrix time	

exposure on development of children: a 3-year follow-up study of China cohort. Environmental Science and Pollution Research International, 26(20), 20780–20786. 10.1007/s11356-019-05362-0			
Liu, D. C., Xu, X. J., Zheng, X. B., Jiang, Y. S., Zhang, J. Q., & Huo, X. (2019). The study of exposure levels of dioxin-like compounds in cord blood of newborns in an e-waste dismantling area in Guangdong Province. Zhonghua Yu Fang Yi Xue Za Zhi [Chinese Journal of Preventive Medicine], 53(4), 365–370. 10.3760/cma.j.issn.0253-9624.2019.04.007	non-English		
Bai, X., Lu, S., Xie, L., Zhang, B., Song, S., He, Y., Ouyang, J., & Zhang, T. (2019). A pilot study of metabolites of organophosphorus flame retardants in paired maternal urine and amniotic fluid samples: potential exposure risks of tributyl phosphate to pregnant women. Environmental Science.Processes & Impacts, 21(1), 124–132. 10.1039/c8em00389k	Flame retardants	PECO	exposure: flame retardants
Grant, K., Goldizen, F. C., Sly, P. D., Brune, M., Neira, M., van den Berg, M., & Norman, R. E. (2013). Health consequences of exposure to e-waste: a systematic review. The Lancet.Global Health, 1(6), 350. 10.1016/S2214-109X(13)70101-3	Mention of risk for pregnant women and their neonates but not a direct study on their exposure and outcomes; possible snowballing resource	PECO	population
Ju, Y., Xu, G., Chen, L., & Shi, J. (2008). Effects of the electronic waste pollution on the levels of thyroid hormone and its receptor expression in the parturient woman and the newborn. Wei Sheng Yan Jiu = Journal of Hygiene Research, 37(5), 536–539.	six weeks post childbirth - thyroid issues	sample matrix time	
Opler, M. G. A., Buka, S. L., Groeger, J., McKeague, I., Wei, C., Factor-Litvak, P., Bresnahan, M., Graziano, J., Goldstein, J. M., Seidman, L. J., Brown, A. S., & Susser, E. S. (2008). Prenatal exposure to lead, delta-aminolevulinic acid, and schizophrenia: further evidence. Environmental Health Perspectives, 116(11), 1586–1590. 10.1289/ehp.10464	Exposure of lead with outcome of schizophrenia focus	PECO	outcome
Opler, M. G. A., Brown, A. S., Graziano, J., Desai, M., Zheng, W., Schaefer, C., Factor-Litvak, P., & Susser, E. S. (2004). Prenatal lead exposure, delta-aminolevulinic acid, and schizophrenia. Environmental Health Perspectives, 112(5), 548–552. 10.1289/ehp.6777	Exposure of lead with outcome of schizophrenia focus	PECO	outcome

Sagiv, S. K., Thurston, S. W., Bellinger, D. C., Amarasiriwardena, C., & Korrick, S. A. (2012). Prenatal exposure to mercury and fish consumption during pregnancy and attention-deficit/hyperactivity disorder-related behavior in children. Archives of Pediatrics & Adolescent Medicine, 166(12), 1123–1131. 10.1001/archpediatrics.2012.1286	Fish consumption and ADHD	PECO	exposure; fish
Stewart, P. W., Reihman, J., Lonky, E. I., Darvill, T. J., & Pagano, J. (2003). Cognitive development in preschool children prenatally exposed to PCBs and MeHg. Neurotoxicology and Teratology, 25(1), 11–22. 10.1016/s0892-0362(02)00320-3	Not related to electronic waste; focus on fish	PECO	exposure: fish
Jacobson, J. L., Jacobson, S. W., & Humphrey, H. E. (1990). Effects of in utero exposure to polychlorinated biphenyls and related contaminants on cognitive functioning in young children. The Journal of Pediatrics, 116(1), 38–45. 10.1016/s0022-3476(05)81642-7	Not related to electronic waste; focus on fish	PECO	exposure: fish
Jacobson, J. L., & Jacobson, S. W. (1996). Intellectual impairment in children exposed to polychlorinated biphenyls in utero. The New England Journal of Medicine, 335(11), 783–789. 10.1056/NEJM199609123351104	Not related to electronic waste; focus on fish	PECO	exposure: fish
Herbstman, J. B., Sjodin, A., Kurzon, M., Lederman, S. A., Jones, R. S., Rauh, V., Needham, L. L., Tang, D., Niedzwiecki, M., Wang, R. Y., & Perera, F. (2010). Prenatal exposure to PBDEs and neurodevelopment. Environmental Health Perspectives, 118(5), 712–719. 10.1289/ehp.0901340	Not related to electronic waste; focus on flame retardants	PECO	exposure: flame retardants
Eskenazi, B., Chevrier, J., Rauch, S. A., Kogut, K., Harley, K. G., Johnson, C., Trujillo, C., Sjodin, A., & Bradman, A. (2013). In utero and childhood polybrominated diphenyl ether (PBDE) exposures and neurodevelopment in the CHAMACOS study. Environmental Health Perspectives, 121(2), 257–262. 10.1289/ehp.1205597	no mention of e-waste	PECO	exposure: no e-waste
Dietrich, K. N., Berger, O. G., Succop, P. A., Hammond, P. B., & Bornschein, R. L. (1993). The developmental consequences of low to moderate prenatal and postnatal lead exposure: intellectual attainment in the Cincinnati Lead Study Cohort following school entry. Neurotoxicology and Teratology, 15(1), 37–44. 10.1016/0892-0362(93)90043-n	Just an exposure study on lead; not directly connected to e-waste	PECO	exposure: no e-waste

Axelrad, D. A., Bellinger, D. C., Ryan, L. M., & Woodruff, T. J. (2007). Dose-response relationship of prenatal mercury exposure and IQ: an integrative analysis of epidemiologic data. *Environmental Health Perspectives, 115*(4), 609–615. 10.1289/ehp.9303	Not related to electronic waste; focus on fish	PECO	exposure: fish
Koopman-Esseboom, C., Morse, D. C., Weisglas-Kuperus, N., Lutkeschipholt, I. J., Van der Paauw, C. G., Tuinstra, L. G., Brouwer, A., & Sauer, P. J. (1994). Effects of dioxins and polychlorinated biphenyls on thyroid hormone status of pregnant women and their infants. Pediatric Research, 36(4), 468–473. 10.1203/00006450-199410000-00009	Not related to electronic waste; focus on fish	PECO	exposure: fish
Wolff, M. S., Engel, S., Berkowitz, G., Teitelbaum, S., Siskind, J., Barr, D. B., & Wetmur, J. (2007). Prenatal pesticide and PCB exposures and birth outcomes. Pediatric Research, 61(2), 243–250. 10.1203/pdr.0b013e31802d77f0	Not related to electronic waste; focus on pesticides	PECO	exposure: pesticides
Rylander, L., Stromberg, U., & Hagmar, L. (2000). Lowered birth weight among infants born to women with a high intake of fish contaminated with persistent organochlorine compounds. Chemosphere, 40(9-11), 1255–1262. 10.1016/s0045-6535(99)00377-x	Focus on fish consumption no e-waste	PECO	exposure: fish
Patandin, S., Koopman-Esseboom, C., de Ridder, M. A., Weisglas-Kuperus, N., & Sauer, P. J. (1998). Effects of environmental exposure to polychlorinated biphenyls and dioxins on birth size and growth in Dutch children. Pediatric Research, 44(4), 538–545. 10.1203/00006450-199810000-00012	Not related to electronic waste; focus on fish	PECO	exposure: fish
Kippler, M., Tofail, F., Gardner, R., Rahman, A., Hamadani, J. D., Bottai, M., & Vahter, M. (2012). Maternal cadmium exposure during pregnancy and size at birth: a prospective cohort study. Environmental Health Perspectives, 120(2), 284–289. 10.1289/ehp.1103711	General analysis on heavy metal impact on pregnant women but no exposure/outcome analysis	PECO	lack of outcome analysis
Harley, K. G., Chevrier, J., Aguilar Schall, R., Sjodin, A., Bradman, A., & Eskenazi, B. (2011). Association of prenatal exposure to polybrominated diphenyl ethers and infant birth weight. American Journal of Epidemiology, 174(8), 885–892. 10.1093/aje/kwr212	general not mention of e-waste	PECO	exposure: no e-waste

Govarts, E., Nieuwenhuijsen, M., Schoeters, G., Ballester, F., Bloemen, K., de Boer, M., Chevrier, C., Eggesbo, M., Guxens, M., Kramer, U., Legler, J., Martinez, D., Palkovicova, L., Patelarou, E., Ranft, U., Rautio, A., Petersen, M. S., Slama, R., Stigum, H., . . . ENRIECO. (2012). Birth weight and prenatal exposure to polychlorinated biphenyls (PCBs) and dichlorodiphenyldichloroethylene (DDE): a meta-analysis within 12 European Birth Cohorts. Environmental Health Perspectives, 120(2), 162–170. 10.1289/ehp.1103767	Not related to electronic waste; focus on pesticides	PECO	exposure: pesticides
Al-Saleh, I., Alsabbahen, A., Shinwari, N., Billedo, G., Mashhour, A., Al-Sarraj, Y., Mohamed, G. E. D., & Rabbah, A. (2013). Polycyclic aromatic hydrocarbons (PAHs) as determinants of various anthropometric measures of birth outcome. The Science of the Total Environment, 444, 565–578. 10.1016/j.scitotenv.2012.12.021	no mention of e-waste	PECO	exposure: no e-waste
Zhang, A., Hu, H., Sanchez, B. N., Ettinger, A. S., Park, S. K., Cantonwine, D., Schnaas, L., Wright, R. O., Lamadrid-Figueroa, H., & Tellez-Rojo, M. M. (2012). Association between prenatal lead exposure and blood pressure in children. Environmental Health Perspectives, 120(3), 445–450. 10.1289/ehp.1103736	Not related to electronic waste; focus on lead	PECO	exposure: no e-waste
Halldorsson, T. I., Rytter, D., Haug, L. S., Bech, B. H., Danielsen, I., Becher, G., Henriksen, T. B., & Olsen, S. F. (2012). Prenatal exposure to perfluorooctanoate and risk of overweight at 20 years of age: a prospective cohort study. Environmental Health Perspectives, 120(5), 668–673. 10.1289/ehp.1104034	Not related to electronic waste; textiles, etc.	PECO	exposure: textiles
Y., Huo, X., Liu, J., Peng, L., Li, W., & Xu, X. (2011). Assessment of cadmium exposure for neonates in Guiyu, an electronic waste pollution site of China. Environmental Monitoring and Assessment, 177(1-4), 343–351. 10.1007/s10661-010-1638-6	increased expression?	insufficient outcome data	
Liu, Q., Cao, J., Li, K. Q., Miao, X. H., Li, G., Fan, F. Y., & Zhao, Y. C. (2009). Chromosomal aberrations and DNA damage in human populations exposed to the processing of electronics waste. Environmental Science and Pollution Research International, 16(3), 329–338. 10.1007/s11356-008-0087-z	not a study on association; can be used as suggested recommendations	PECO	lack of outcome analysis

Wu, K., Xu, X., Liu, J., Guo, Y., Li, Y., & Huo, X. (2010). Polybrominated diphenyl ethers in umbilical cord blood and relevant factors in neonates from Guiyu, China. Environmental Science & Technology, 44(2), 813–819. 10.1021/es9024518	PBDE not heavy metals	PECO	exposure: PBDE
Guo, Y., Huo, X., Wu, K., Liu, J., Zhang, Y., & Xu, X. (2012). Carcinogenic polycyclic aromatic hydrocarbons in umbilical cord blood of human neonates from Guiyu, China. The Science of the Total Environment, 427-428, 35–40. 10.1016/j.scitotenv.2012.04.007	NA		
Guo, Y., Huo, X., Li, Y., Wu, K., Liu, J., Huang, J., Zheng, G., Xiao, Q., Yang, H., Wang, Y., Chen, A., & Xu, X. (2010). Monitoring of lead, cadmium, chromium and nickel in placenta from an e-waste recycling town in China. The Science of the Total Environment, 408(16), 3113–3117. 10.1016/j.scitotenv.2010.04.018	PAHs not heavy metals	PECO	Exposure: PAHs
Chen, A., Dietrich, K. N., Huo, X., & Ho, S. (2011). Developmental neurotoxicants in e-waste: an emerging health concern. Environmental Health Perspectives, 119(4), 431–438. 10.1289/ehp.1002452	general analysis of harm based on other studies	PECO	lack of outcome analysis
Heacock, M., Kelly, C. B., Asante, K. A., Birnbaum, L. S., Bergman, A. L., Brune, M., Buka, I., Carpenter, D. O., Chen, A., Huo, X., Kamel, M., Landrigan, P. J., Magalini, F., Diaz-Barriga, F., Neira, M., Omar, M., Pascale, A., Ruchirawat, M., Sly, L., . . . Suk, W. A. (2016). E-Waste and Harm to Vulnerable Populations: A Growing Global Problem. Environmental Health Perspectives, 124(5), 550–555. 10.1289/ehp.1509699	not an outcome study --> discussion analysis	PECO	lack of outcome analysis
Wang, J., & Guo, X. (2006). Impact of electronic wastes recycling on environmental quality. Biomedical and Environmental Sciences : BES, 19(2), 137–142.	general analysis on environmental quality from e-waste	PECO	lack of outcome analysis
Luo, Q., Wong, M., & Cai, Z. (2007). Determination of polybrominated diphenyl ethers in freshwater fishes from a river polluted by e-wastes. Talanta, 72(5), 1644–1649. 10.1016/j.talanta.2007.03.012	fish exposure/levels due to e-waste	PECO	exposure: fish
Li, H., Yu, L., Sheng, G., Fu, J., & Peng, P. (2007). Severe PCDD/F and PBDD/F pollution in air around an electronic waste dismantling area in China. Environmental Science & Technology, 41(16), 5641–5646. 10.1021/es0702925	Study finding exact levels of e-waste related toxins but no association with pregnant women and their neonates	PECO	population

Yekeen, T. A., Xu, X., Zhang, Y., Wu, Y., Kim, S., Reponen, T., Dietrich, K. N., Ho, S., Chen, A., & Huo, X. (2016). Assessment of health risk of trace metal pollution in surface soil and road dust from e-waste recycling area in China. Environmental Science and Pollution Research International, 23(17), 17511–17524. 10.1007/s11356-016-6896-6	No connection to pregnant women or their neonates	PECO	population
Wong, M. H., Wu, S. C., Deng, W. J., Yu, X. Z., Luo, Q., Leung, A. O. W., Wong, C. S. C., Luksemburg, W. J., & Wong, A. S. (2007). Export of toxic chemicals - a review of the case of uncontrolled electronic-waste recycling. Environmental Pollution (Barking, Essex : 1987), 149(2), 131–140. 10.1016/j.envpol.2007.01.044	looks at air, soil, etc., all contamination from e-waste but not effect association	PECO	population
Huo, X., Zheng, X. B., Liu, Q., Zhang, T., Wang, Q. H., & Xu, X. J. (2019). Impact of informal e-waste recycling on human health. Zhonghua Yu Fang Yi Xue Za Zhi [Chinese Journal of Preventive Medicine], 53(4), 426–432. 10.3760/cma.j.issn.0253-9624.2019.04.020	non-English		
Zhang, R., Chen, L. P., & Chen, W. (2019). Hazards and challenges from global e-waste pollution. Zhonghua Yu Fang Yi Xue Za Zhi [Chinese Journal of Preventive Medicine], 53(4), 342–344. 10.3760/cma.j.issn.0253-9624.2019.04.002	general analysis on overall hazard	PECO	lack of outcome analysis
Krishnamoorthy, Y., M, V., Sakthivel, M., & Sarveswaran, G. (2018). Emerging public health threat of e-waste management: global and Indian perspective. Reviews on Environmental Health, 33(4), 321–329. 10.1515/reveh-2018-0021	Systematic review of overall production and harm of e-waste with an overview of potential health effects. No direct association between exposure and outcomes in pregnant women and their neonates.	PECO	lack of outcome analysis
Heacock, M., Kelly, C. B., & Suk, W. A. (2016). E-waste: the growing global problem and next steps. Reviews on Environmental Health, 31(1), 131–135. 10.1515/reveh-2015-0045	Systematic review of overall production and harm of e-waste with an overview of potential health effects. No direct association between exposure and outcomes in pregnant women and their neonates.	PECO	lack of outcome analysis
Magalini, F. (2016). Global challenges for e-waste management: the societal implications. Reviews	policy considerations	PECO	lack of outcome analysis

on Environmental Health, 31(1), 137–140. 10.1515/reveh-2015-0035			
Hossain, M. S., Al-Hamadani, S. M. Z. F., & Rahman, M. T. (2015). E-waste: A Challenge for Sustainable Development. Journal of Health & Pollution, 5(9), 3–11. 10.5696/2156-9614-5-9.3	Good source for evidence of causation; not a direct exposure/outcome study	PECO	lack of outcome analysis
Perkins, D. N., Brune Drisse, M., Nxele, T., & Sly, P. D. (2014). E-waste: a global hazard. Annals of Global Health, 80(4), 286–295. 10.1016/j.aogh.2014.10.001	Good source for evidence of causation; not a direct exposure/outcome study	PECO	lack of outcome analysis
Wong, C. S. C., Wu, S. C., Duzgoren-Aydin, N. S., Aydin, A., & Wong, M. H. (2007). Trace metal contamination of sediments in an e-waste processing village in China. Environmental Pollution (Barking, Essex : 1987), 145(2), 434–442. 10.1016/j.envpol.2006.05.017	sediment samples for e-waste exposure to the environment	PECO	population
Yu, X. Z., Gao, Y., Wu, S. C., Zhang, H. B., Cheung, K. C., & Wong, M. H. (2006). Distribution of polycyclic aromatic hydrocarbons in soils at Guiyu area of China, affected by recycling of electronic waste using primitive technologies. Chemosphere, 65(9), 1500–1509. 10.1016/j.chemosphere.2006.04.006	soil related	PECO	population
Wang, D., Cai, Z., Jiang, G., Leung, A., Wong, M. H., & Wong, W. K. (2005). Determination of polybrominated diphenyl ethers in soil and sediment from an electronic waste recycling facility. Chemosphere, 60(6), 810–816. 10.1016/j.chemosphere.2005.04.025	soil related	PECO	population
Leung, A. O. W., Luksemburg, W. J., Wong, A. S., & Wong, M. H. (2007). Spatial distribution of polybrominated diphenyl ethers and polychlorinated dibenzo-p-dioxins and dibenzofurans in soil and combusted residue at Guiyu, an electronic waste recycling site in southeast China. Environmental Science & Technology, 41(8), 2730–2737. 10.1021/es0625935	Related to town but on amounts found in soil concentrations not within pregnant women	PECO	population
Deng, W. J., Zheng, J. S., Bi, X. H., Fu, J. M., & Wong, M. H. (2007). Distribution of PBDEs in air particles from an electronic waste recycling site compared with Guangzhou and Hong Kong, South China. Environment International, 33(8), 1063–1069. 10.1016/j.envint.2007.06.007	air samples	PECO	population
Leung, A. O. W., Duzgoren-Aydin, N. S., Cheung, K. C., & Wong, M. H. (2008). Heavy metals	Related to town but on amounts found in dust	PECO	population

concentrations of surface dust from e-waste recycling and its human health implications in southeast China. Environmental Science & Technology, 42(7), 2674–2680. 10.1021/es071873x	concentrations not within pregnant women		
Guo, Y., Huang, C., Zhang, H., & Dong, Q. (2009). Heavy metal contamination from electronic waste recycling at Guiyu, Southeastern China. Journal of Environmental Quality, 38(4), 1617–1626. 10.2134/jeq2008.0398	Related to town but on amounts found in water concentrations not within pregnant women	PECO	population
Landrigan, P. J., Raps, H., Cropper, M., Bald, C., Brunner, M., Canonizado, E. M., Charles, D., Chiles, T. C., Donohue, M. J., Enck, J., Fenichel, P., Fleming, L. E., Ferrier-Pages, C., Fordham, R., Gozt, A., Griffin, C., Hahn, M. E., Haryanto, B., Hixson, R., . . . Dunlop, S. (2023). Correction: The Minderoo-Monaco Commission on Plastics and Human Health. Annals of Global Health, 89(1), 71. 10.5334/aogh.4331	Unrelated, abstract was misleading	PECO	
